# Molecular Mechanism of the Therapeutic Effect of Peach Blossom against Constipation: An Exploratory Study Based on Network Pharmacology Analysis and Molecular Docking Verification

**DOI:** 10.1155/2023/8577485

**Published:** 2023-04-06

**Authors:** Bo Ma, Jinyu Ning, Fengyun Wang, Huiling Zheng, Liang Han

**Affiliations:** ^1^Department of Gastroenterology, The East Division of the First Affiliated Hospital of Sun Yat-sen University, Guangzhou, China; ^2^School of Traditional Chinese Medicine, Guangdong Pharmaceutical University, Guangzhou, China; ^3^School of Health, Guangdong Pharmaceutical University, Guangzhou, China

## Abstract

**Objective:**

The aim of the study is to verify the active ingredients of peach blossom and to explore the molecular mechanisms of their therapeutic effects against constipation through network pharmacology and molecular docking analysis.

**Methods:**

The potential active ingredients of peach blossom were identified from published literature and the BAT-TCM database, and their potential targets were predicted using the SwissTargetPrediction and PharmMapper platforms. In addition, targets related to constipation were retrieved using OMIM, DrugBank, GeneCards, TTD, and DisGeNET databases. The intersection of drug targets and disease targets was considered as the potential targets of peach blossom in the treatment of constipation. The STRING platform was used to construct a protein interaction network. Gene ontology (GO) functional analysis and KEGG pathway enrichment analysis were performed on key targets using the DAVID database. Molecular docking verification between the active ingredients of peach blossom and the targets was conducted using AutoDock software.

**Results:**

A total of 33 active ingredients of peach blossom and 185 corresponding targets were identified, and 88 intersection targets were obtained after Venny mapping. These 33 active ingredients (including naringenin, aromadendrin, and cordycepin) in peach blossom may play a role in the treatment of constipation by regulating signaling pathways through targets such as EGFR, VEGFA, ESR1, GSTP1, and PTGS2.

**Conclusion:**

A variety of active ingredients of peach blossom regulate multiple signaling pathways by acting on targets, which reflects the characteristic of “multiple ingredients-multiple targets-multiple pathways,” thereby playing a role in the treatment of constipation.

## 1. Introduction

Constipation is a common gastrointestinal disease characterized by a prolonged or shortened defecation cycle, difficult defecation, and dry stool. In some conditions, the stool is not hard, but the process of defecation is not smooth though there is a desire to defecate [1]. Constipation can be divided into three subtypes: colonic slow transit constipation, outlet obstructive constipation, and functional defecation disorders [2]. According to the “Guidelines for the diagnosis and treatment of chronic constipation in China,” the etiology of constipation is divided into three categories: functional diseases, organic diseases, and drug-induced [3]. With the recent improvement of living standards in China and the associated lifestyle changes (such as changes in diet structure and increased work pressure), the prevalence of constipation in the general population in China is estimated from 3.6% to 12.9% [4], and the prevalence is increased rapidly in recent years.

Currently, the main strategy for the treatment of constipation is the use of laxatives, including bulk laxatives (e.g., psyllium, methylcellulose, calcium polycarbonate, and wheat dextrin [5]), osmotic laxatives (e.g., Polyethylene glycol, sorbitol, and glycerol [6]), and irritant laxatives (e.g., senna and bisacodly [7]). Although these drugs are effective in the short term, there are many adverse reactions, and the constipation is liable to relapse or even aggravate after drug withdrawal. Traditional Chinese medicine (TCM) offers unique advantages in the treatment of constipation. The holistic concept of TCM entails performing dialectic analysis, adjusting the balance of yin and yang in the human body, and treatment of both the symptoms and the root cause of the disease. TCM remedies for constipation are safe and have long-term efficacy [8] and can complement the treatment of western medicine. The top 10 TCM for constipation clinical treatment [9] are rhubarb*, Fructus aurantii, Magnolia officinalis, Atractylodes, Fructus fructus, Cistanche, Astragalus,* peach kernels*, and glycyrrhiza*. Both alone and in combination with other TCM applications, obvious efficacy without toxic side effects has been observed. In addition, it has been reported that the therapeutic effect of TCM on antipsychogenic constipation was more effective than that of western medicines, such as phenolphthalein and glycerin enema [10].

Peach blossoms are the flowers of rosaceous plants *Rosaceae Prunus persica* (L.) Batsch or *Prunus davidiana* (Carr.) Franch. The blooming of flowers precedes the growth of leaves, and the flowers are harvested between March and April [11]. Peach blossom is bitter in taste and neutral in nature and belongs to the heart, liver, and large intestine meridians. It promotes diuresis and defecation, improves blood circulation, and alleviates blood stasis. It is mainly used in the treatment of constipation, edema, dysuria, phlegm retention, amenorrhea, and mania [12]. Its use has been recorded in many TCM classics including “Tang Materia Medica,” “Compendium of Materia Medica,” and “Collected Works of Materia Medica.” Weng et al. [13] used “Shengdi Baizhu Taohua Decoction” to treat 116 cases of senile habitual constipation. After taking the medicine, approximately 95.69% of patients had smooth bowel movements, and there were no side effects such as abdominal pain. Peach blossom has the effects of slowing down and eliminating accumulation and catharsis, and promoting water flow. It does not cause irritation of the intestinal wall and does not elicit abdominal pain [14]. In addition, a previous study found that ethyl acetate extract from peach blossom can promote gastric emptying, intestinal motility, and secretion of gastrointestinal hormones in rats [15]. In clinical practice, we have observed good clinical effects of peach blossom decoction when used as an adjuvant treatment for constipation (data not shown). Although peach blossom has been used as a natural medicine to promote gastrointestinal motility for many years, the underlying molecular mechanisms of its therapeutic effect against constipation are yet to be elucidated. Network pharmacology and molecular docking analysis not only provide ideas for the research and development of TCM but also provide a theoretical basis for clinical application for constipation treatment. The underlying mechanism of TCM for constipation treatment could be that the active ingredients from TCM act on targeted genes such as AKT1, thus playing therapeutic roles by regulating cancer, phosphatidylinositol 3-kinase-protein kinase B and p53 signaling pathways [16].

In this study, an “active ingredient-target-signaling pathway” network was constructed based on the network pharmacology analysis, and the potential molecular mechanisms of the therapeutic effect of peach blossom against constipation were explored by molecular docking analysis and verification. Our findings may provide a theoretical basis for the clinical application of peach blossom in the treatment of constipation, and provide novel insights for further research on the mechanisms of peach blossom.

## 2. Materials and Methods

### 2.1. Screening of Active Ingredients of Peach Blossom and Target Prediction

The chemical ingredients of peach blossom were retrieved from the BATMAN-TCM database (https://bionet.ncpsb.org/batman-tcm/) [17], and the relevant literature was searched in the CNKI, Wanfang, and PubMed databases [18–22] to supplement the results of the active ingredients of peach blossoms. The SDF molecular structures of the active ingredients were downloaded from PubChem (https://pubchem.ncbi.nlm.nih.gov). The SwissADME database (https://www.swissadme.ch/) [23] was searched, and the ingredients satisfying the Lipinski rule [24] and high gastrointestinal (GI) absorption were screened as active ingredients for subsequent analysis. The potential targets of active ingredients were predicted using the SwissTargetPrediction [25] and PharmMapper [26] platforms.

### 2.2. Collection of Disease Targets

The OMIM (Online Mendelian Inheritance in Man, https://www.omim.org/) [27], DrugBank (https://www.drugbank.ca/) [28], GeneCards (https://www.genecards.org/) [29], TTD (Therapeutic Target Database, https://db.idrblab.net/ttd/) [30], and DisGeNET (https://www.disgenet.org/) [31] databases were used for collecting the targets of disease. The target species was selected as “*Homo sapiens*,” and the search was conducted using “constipation” as the keyword, and the targets closely related to constipation were screened. The obtained targets were standardized using the UniProt database [32].

### 2.3. Venn Analysis of the Potential Targets of Peach Blossom in the Treatment of Constipation

The screened targets of active ingredients and disease were uploaded to the Venny 2.1 online tool website (https://bioinfogp.cnb.csic.es/tools/venny/index.html). A Venn diagram was constructed to obtain the intersection targets, which were the potential targets of peach blossom in the treatment of constipation.

### 2.4. Construction of the “Active Ingredient-Target” Network

Cytoscape 3.8.0 software [33] was used to construct and analyze the relationship network between the potential active ingredients and the targets of peach blossom in the treatment of constipation. In the network, the active components and targets of the peach blossom were represented by nodes, and the relationship between active components and targets was represented by edges.

### 2.5. Construction of the Protein-Protein Interaction (PPI) Network

The drug-disease intersection targets were uploaded to the STRING (https://string-db.org/) database [34] to construct the PPI network. The species was limited to “*Homo sapiens*,” and “Medium confidence = 0.400” was set to obtain the PPI network. The “.tsv” format file was downloaded and imported into the Cytoscape 3.8.0 software. The plug-in CytoNCA [35] was used to perform a topology analysis of each node in the intersection network. The parameters such as degree, betweenness, and closeness between each node were adjusted to obtain the most closely related key targets.

### 2.6. Gene Function Annotation and Pathway Enrichment Analysis

Using the DAVID database (https://david.ncifcrf.gov/) [36], Gene Ontology (GO) functional annotation and Kyoto Encyclopedia of Genes and Genomes (KEGG) pathway analyses were performed for the key drug-disease targets. *P* < 0.05 was set as the threshold for screening. The GO and KEGG enrichment results were plotted into histograms and bubble charts using the bioinformatics online platform (https://www.bioinformatics.com.cn/).

### 2.7. Construction and Analysis of the “Active Ingredient-Target-Signaling Pathway” Network

The potential active ingredients of peach blossom, their molecular targets, and the potential signaling pathways mediating the therapeutic effect of peach blossom against constipation were imported into Cytoscape 3.8.0 software to construct a visual network. The Network Analyzer plug-in was used for network topology analysis to screen important active ingredients and action targets.

### 2.8. Molecular Docking Verification

The 3D structures of MAPK1, EGFR, VEGFA, PTGS2, and ESR1 proteins were downloaded from the RCSB PDB database (https://www.rcsb.org/), and the SDF structures of the active ingredients of peach blossom were converted into “mol2” format files using OpenBabel software. The AutoDock Tools-1.5.6 software was used to dewater and hydrogenate the proteins, and save it as “PDBQT” format. Then, the processed protein was combined with its corresponding active ingredient by AutoDock Tools-1.5.6 software to obtain the affinity. Affinity < 0 indicated that the ligand and the receptor can spontaneously bind, and the conformation with the lowest affinity was the optimal conformation. The PyMOL software was used to conduct a visualization analysis of the molecular docking results.

## 3. Results

### 3.1. Active Ingredients and the Targets of Peach Blossom

According to the results of BATMAN-TCM database analysis and literature reports, a total of 79 ingredients were identified, and the candidate active ingredients with high gastrointestinal absorption were screened. Eventually, 33 active ingredients were selected (Table 1). The SDF structure information of the 33 ingredients were downloaded on the PubChem website, and the corresponding targets were predicted through the Swiss Prediction platform. The top 10 targets in the probability ranking were retained. If the target information of the ingredient was not included in the Swiss Prediction website, the PharmMapper was used for supplementation. After deleting the duplicate targets, a total of 185 potential targets were obtained.

### 3.2. Intersection of Peach Blossom Targets and Constipation Targets

**Figure 1:**
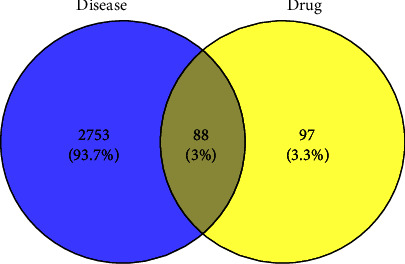
Analysis of intersection targets of active ingredients and constipation.

The disease-related databases were retrieved and the targets were screened according to score and species. Among them, 22, 59, 2778, 9, and 295 targets were obtained in OMIM, DrugBank, GeneCards, TTD, and DisGeNET databases, respectively. A total of 2841 related targets were obtained after the summation and deletion of the duplicate targets. The screened targets of drug and disease were uploaded to the Venny 2.1 website, and a Venn diagram was generated. Eventually, 88 intersecting targets were extracted for further analysis (Figure 1).

### 3.3. Construction and Analysis of the “Active Ingredients-Targets” Network

**Figure 2:**
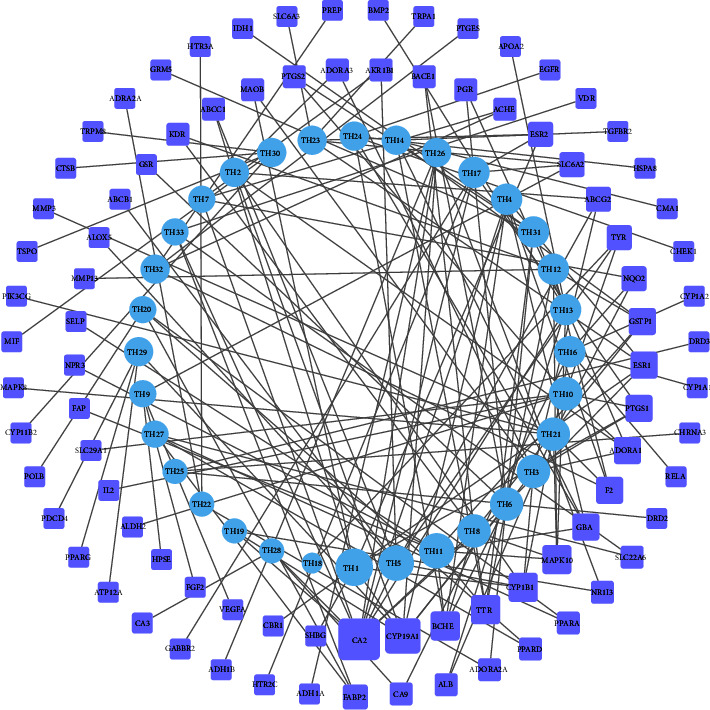
Network diagram of “active ingredients-targets” of peach blossom in the treatment of constipation. The blue circle node represents the active ingredient, and the purple rectangle node represents the action targets of the active ingredient.

The Cytoscape 3.8.0 was used to draw and analyze the network relationship diagram of the potential active ingredients of peach blossom and intersection targets in the treatment of constipation. As shown in Figure 2, the network consisted of 120 nodes and 167 edges. The size of a node in the diagram represented the corresponding value of a degree, and the degree value was the number of connecting edges. The larger the degree value, the stronger the pivotal role of the node in the network, the more biological functions involved, and the greater the biological importance. According to the value of degree, the top 5 compounds were naringenin, kaempferol, multiflorin A, kaempferol-3-O-rutinoside nicotiflorin, and (-)-catechin, which had 9, 8, 8, 7, and 7 corresponding targets, respectively, with relatively strong activity. The top 5 targets were CA2, CYP19A1, BCHE, TTR, and CYP1D1.

### 3.4. Construction of the PPI Network and Screening of Key Targets

**Figure 3:**
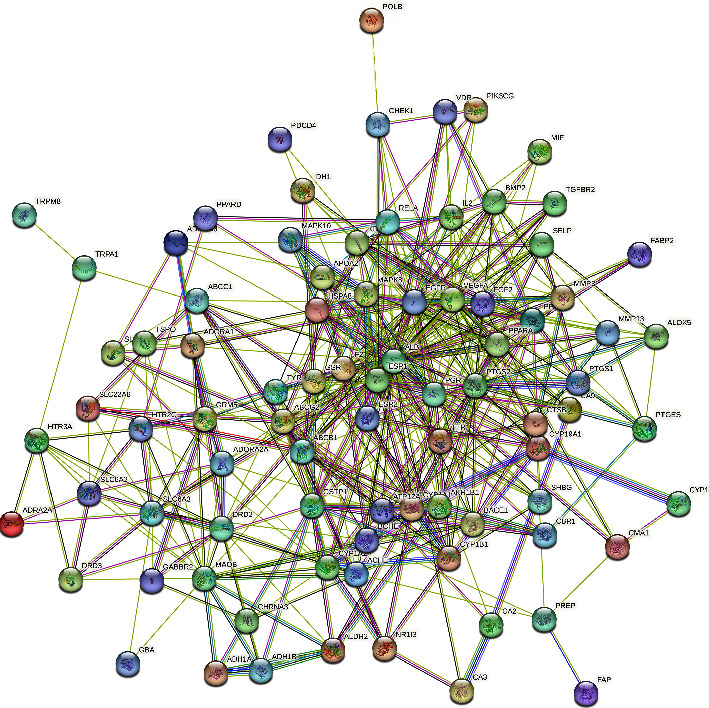
Interaction network of intersection target proteins.

**Figure 4:**
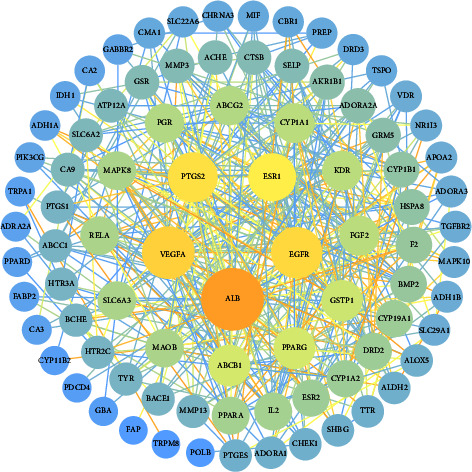
PPI-protein interaction topology analysis network. The size and color of the node represents the value of the degree. The node changes from small to large, the color changes from blue to orange, and the corresponding value of the degree changes from small to large. The thickness and color of the edge represents the value of the overall score. The edge changes from thin to thick, the color changes from blue to orange, and the corresponding overall score value changes from small to large.

**Figure 5:**
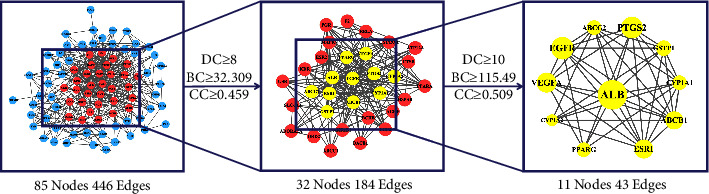
Screening of key targets.

The STRING platform was used to construct the interaction relationship of the intersection target proteins, and the PPI-protein interaction network diagram was obtained. As shown in Figure 3, a total of 88 interaction nodes and 446 edges were obtained in the network. The “.TSV” format file was downloaded and imported into Cytoscape 3.8.0 software to obtain a visualized network diagram. The targets that were not connected to other targets were deleted (Figure 4). Network topology analysis was conducted using the CytoNCA, a Cytoscape 3.8.0 software plug-in. The degree, betweenness, and closeness parameters were adjusted to screen the targets. Those with a value larger than the median value were retained. After screening using twice the median value as a threshold, 11 key targets were obtained (Figure 5). According to the value of a degree in the PPI network, the top 5 proteins were ALB (52), VEGFA (37), EGFR (35), PTGS2 (33), and ESR1 (30).

### 3.5. GO and KEGG Enrichment Analysis

**Figure 6:**
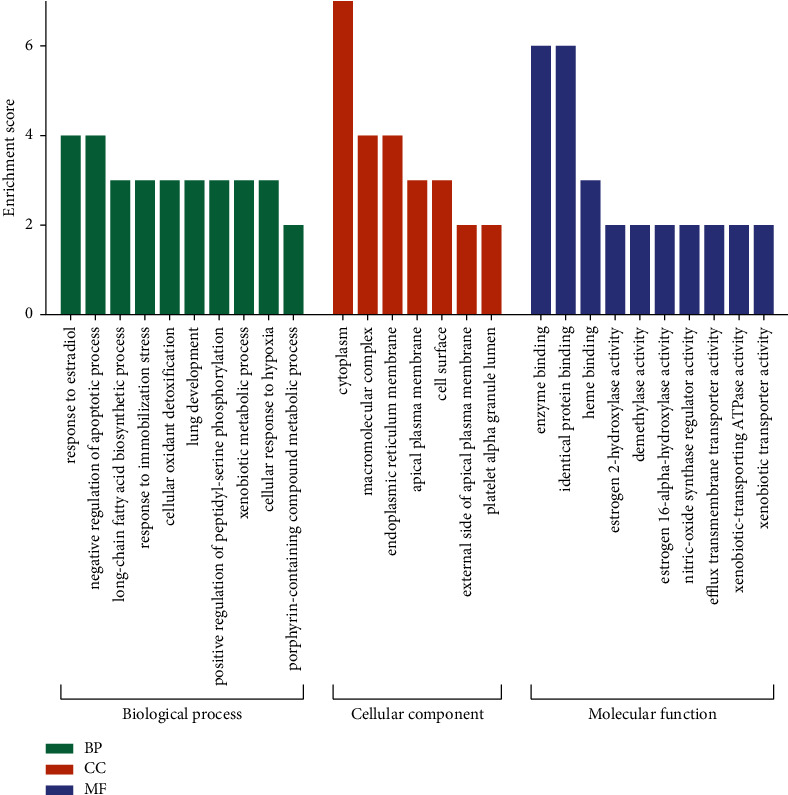
GO enrichment analysis of key targets.

**Figure 7:**
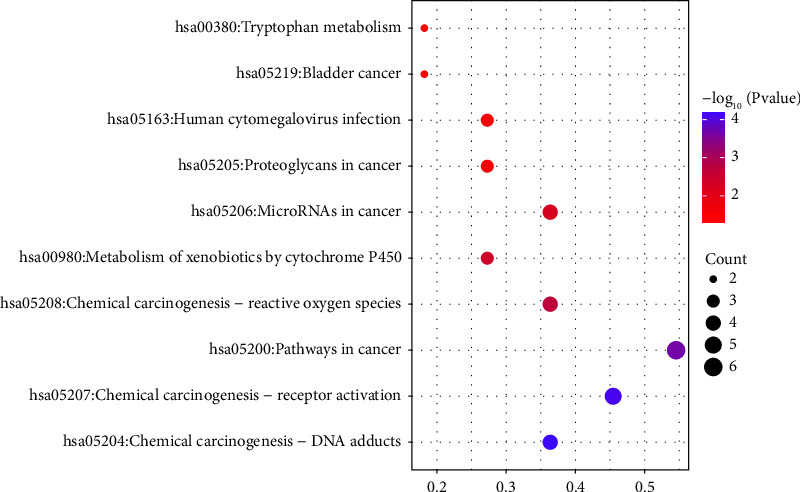
KEGG pathway enrichment analysis of key targets.

The David platform was used for enrichment analysis of the above 11 key targets, including the biological process (BP), cellular component (CC), and molecular function (MF) of GO, as well as the KEGG pathway. With *P* < 0.05 as the screening criterion, the top 10 entries in BP, CC, and MF were selected to draw a histogram (Figure 6). A bubble chart was drawn for the KEGG pathways with the top 10 ranking of *P* value (Figure 7). The color of the bubbles from purple to red represented the *P* value from small to large. The smaller the *P* value, the stronger was the significance. The size of the bubble represented the count of genes in the pathway, and the horizontal axis represented the ratio of the pathway genes to the total input genes.

The main biological processes involved in the treatment of constipation by peach blossom include negative regulation of the apoptotic process, response to estradiol, long-chain fatty acid biosynthetic process, response to immobilization stress, xenobiotic metabolic process, and cellular response to hypoxia. The molecular functions involved were mainly enzyme binding, identical protein binding, heme binding, nitric-oxide synthase regulator activity, and estrogen 2 -hydroxylase activity.

The KEGG signaling pathway was mainly enriched in tumor-related pathways, including chemical carcinogenesis-receptor activation, chemical carcinogenesis-DNA adducts, and chemical carcinogenesis-activity oxygen species. It also affected the metabolic pathways, such as the metabolism of xenobiotics by cytochrome P450 and tryptophan metabolism.

### 3.6. Construction and Analysis of the “Active Ingredient-Target-Signaling Pathway” Network

**Figure 8:**
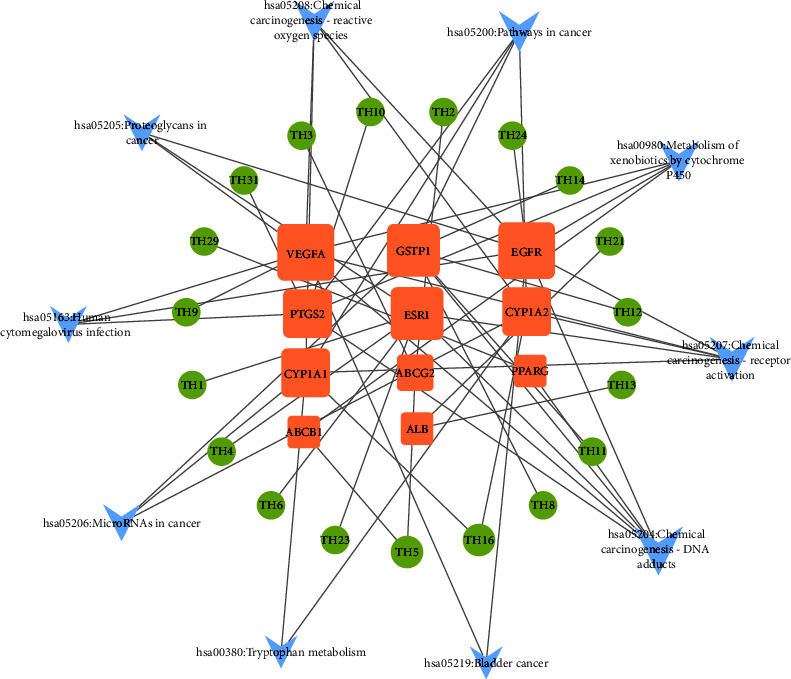
“Active ingredient-target-signaling pathway” network diagram of peach blossom in the treatment of constipation. The orange-yellow rectangular nodes represent targets, the green circular nodes represent active ingredients, and the blue arrows represent pathways.

Cytoscape 3.8.0 was used to draw a network diagram for the top 10 pathways in KEGG analysis and their corresponding active ingredients and targets, and visualization analysis was performed (Figure 8). The network diagram had a total of 40 nodes and 57 edges. The size of the node represented the corresponding value of a degree, and the degree value indicated the number of connected edges. In the diagram, the main active ingredients of peach blossom, such as cordycepin, benzyl-*β*-D-glucopyranoside, naringenin, aromadendrin, and (-)-catechin, were found to act on the important signaling pathways mediating the therapeutic effect of peach blossom against constipation by binding to targets such as EGFR, VEGFA, and ESR1. Among these, the top 5 targets with a degree ≥2 times of the median were EGFR, VEGFA, ESR1, GSTP1, and PTGS2. These findings suggested that these proteins are potential core targets of peach blossom in the treatment of constipation. The active ingredients of peach blossom act on multiple targets and different pathways interact with each other through multiple common targets, thereby playing a synergistic role in the treatment of constipation.

### 3.7. Molecular Docking Analysis

**Figure 9:**
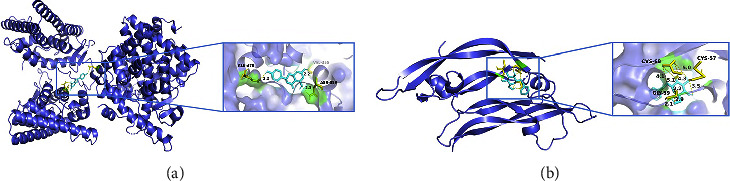
Molecular docking of core targets and active ingredients of peach blossom. The cyan stick-like structure is the compound, the purple ribbon structure is the protein, the yellow part is the stick-like structure of amino acid residues connected to the compounds, and the green part is the schematic illustration of the structure of amino acid residues connected to the compound. The number indicates the length of the hydrogen bond of the compound.

Molecular docking was performed for the five core targets (including EGFR, VEGFA, ESR1, GSTP1, and PTGS2) and their corresponding compounds. It is generally believed that the lower the affinity, the greater is the possibility of the target protein binding to the compound, and the more stable is the binding conformation. The results of molecular docking between the active ingredients of peach blossom and the core targets are shown in Table 2. The targets and compounds with a docking affinity less than −5 kcal/mol are shown in Figure 9.

## 4. Discussion

Peach blossom can not only be used as a laxative, as recorded by Shizhen Li in “Compendium of Materia Medica (本草纲目),” but also for dietary therapy to treat dry feces and intestinal obstruction ever since [37]. As recorded in “Qian Jin Fang (千金方),” consuming a spoonful of peach blossom with water could treat constipation. According to “Taiping Shenghui Fang (太平圣惠方),” ravioli made of peach blossom could treat dry feces, stuffy intestines, and abdominal distension and pain peach blossom has a great laxative effect. However, its active ingredients, effect target, and potential mechanisms are still not clear. Molecular docking can be used to virtually screen out active compounds in drugs by scoring the combination of active ingredients and effect target, and analyze by predicting their binding ways and affinities [38]. This study was based on network pharmacology and molecular docking analysis, exploring the potential mechanisms of peach blossom treatment of constipation.

A search conducted on the BATMAN-TCM database identified 79 potential active ingredients of peach blossom. After the literature review and excluding the ingredients with low gastrointestinal absorption rate, a total of 33 active ingredients were included in the study; most of these were flavonoids and alkaloids, including naringenin, hesperetin, aromadendrin, and persicogenin. A previous study [39] found that both naringenin and hesperetin can significantly promote small bowel movements in normal mice, and the effect was stronger when the two agents were used together. Kaempferol has been shown to play a regulatory role in the intervention of diarrheal irritable bowel syndrome (IBS-D) and is one of the main compounds of “large-headed atractylodes decoction (参苓白术散)” for IBS-D treatment [40]. “Active ingredient-target-signaling pathway” network analysis showed that peach blossom has multiingredient, multitarget, and multipathway signaling characteristics in the treatment of constipation. EGFR, VEGFA, ESR1, GSTP1, and PTGS2 were identified as the core targets of peach blossom; of these, EGFR, VEGFA, ESR1, and PTGS2 were the targets predicted by both PPI network analysis and “active ingredient-target-signaling pathway” analysis. During the molecular docking analysis, naringenin and benzyl beta-D-glucopyranoside showed the best binding effects to the ESR1 target and VEGFA target, respectively. The *ESR1* gene encodes the estrogen receptor ER*α*. Studies have demonstrated upregulation of the expression of ER*α* in the intestinal mucosa of patients with irritable bowel syndrome, resulting in the dysregulation of estrogen-mediated local immune responses, which may be related to the pathogenesis of irritable bowel syndrome [41]. Vascular endothelial growth factor (VEGF) specifically promotes vascular endothelial cell proliferation, migration, and angiogenesis, inhibits cell apoptosis, and induces increased vascular permeability [42]. VEGFA is the most important subtype exerting biological functions in the VEGF family, which has been widely studied in inflammation and cancers [43]. VEGFA is overexpressed in colitis tissues [44].

In this study, GO enrichment analysis found that the main molecular functions regulated by peach blossom in the treatment of constipation were nitric-oxide synthase (NOS) regulatory activity, heme binding, and efflux transmembrane transporter activity. NO plays an important role in regulating gastrointestinal motility and is the main inhibitory neurotransmitter affecting intestinal motility. NOS is a key enzyme for the production of endogenous NO and exists in the gastrointestinal tissues from the esophagus to the internal anal sphincter [45]. Fan et al. [46] found that high-dose Zhizhu Tongbian Decoction affected intestinal motility by reducing the expression of NOS mRNA in the colon of rats, thereby significantly improving the intestinal transmission and enteric neurotransmission system of slow-transit constipation rats. Chen et al. [47] found that the decrease in distribution and expression of heme oxygenase-2 in the colon tissues of rat may be one of the causes of colonic movement disorder in diabetes. In recent years, studies have demonstrated the expression of a large number of aquaporins (AQPs) in the intestine, which can maintain the homeostasis of the internal and external environment of cells. In the study by Qian et al. [48], Tongbian granules were found to significantly improve the defecation function of rats with slow transit constipation, which may be achieved by regulating the absorption and secretion of water by downregulating the expression of AQP3 and AQP8.

KEGG pathway enrichment analysis revealed the potential involvement of several pathways in mediating the therapeutic effect of peach blossom against constipation including cancer signaling pathways, metabolism of cytochrome P450 to exogenous substances, and tryptophan metabolism. Cytochrome P450 (CYP450) is a superfamily of enzymes that exists widely in the body. CYP3A is a subenzyme with the highest content in the CYP450 enzyme system, which mainly exists in the intestine and liver of animals. Approximately, 60% of drugs are catalyzed by the CYP3A enzyme system to complete the metabolic process [49]. Wang et al. [50] found that modulating the tryptophan pathway to regulate the L-tryptophan and serotonin levels can enhance the gastrointestinal motility, suggesting a direct relationship between the tryptophan metabolic pathway and gastrointestinal motility.

Studies have shown that Chaihu Shugan powder can significantly inhibit the apoptosis of gastric Cajal interstitial cells and effectively improve the gastrointestinal function [51]. Similarly, the effects of peach blossom in the treatment of constipation may be achieved by regulating the biological processes including cell apoptosis and body metabolism, by affecting the functions of molecules such as heme, NOS, and transmembrane transporters, and by participating in the metabolism of exogenous substances by cytochrome P450, tryptophan metabolism, and other pathways, thereby regulating gastrointestinal motility. According to the KEGG analysis of core targets, the processes involve chemical carcinogenesis-receptor activation, chemical carcinogenesis-DNA adducts, bladder cancer, and other cancer-related pathways.

## 5. Conclusion

In this study, we performed network pharmacology analysis to systematically analyze the potential active ingredients, effect targets, and signaling pathways mediating the therapeutic effect of peach blossom against constipation. A total of 33 active ingredients (including naringenin, aromadendrin, and cordycepin) in peach blossoms were identified, which might play a role in the treatment of constipation by regulating signaling pathways through targeted genes such as EGFR, VEGFA, ESR1, GSTP1 and PTGS2, which reflects the characteristic of “multi-ingredient-multi-target-multi-pathway” therapy. This study lays a foundation and provides a theoretical basis for further research on the mechanism of the laxative effect of peach blossom.

## Figures and Tables

**Table 1:** Active ingredients of peach blossom.

ID	CAS number	Molecule's name	Chemical formula	Structure
TH1	480-41-1	Naringenin	C_15_H_12_O_5_	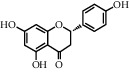
TH2	520-33-2	Hesperetin	C_16_H_14_O_6_	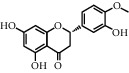
TH3	28590-40-1	Persicogenin	C_17_H_16_O_6_	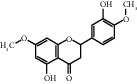
TH4	480-20-6	Aromadendrin	C_15_H_12_O_6_	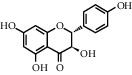
TH5	520-18-3	Kaempferol	C_15_H_10_O_6_	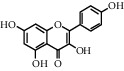
TH6	18829-70-4	(−)-Catechin	C_15_H_14_O_6_	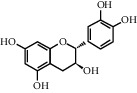
TH7	480-10-4	Kaempferol-3-O-glucoside	C_21_H_20_O_11_	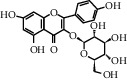
TH8	17650-84-9	Kaempferol-3-O-rutinoside	C_27_H_30_O_15_	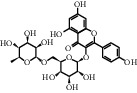
TH9	4304-12-5	Benzyl beta-D-glucopyranoside	C_13_H_18_O_6_	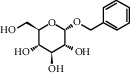
TH10	6807-83-6	Trifolirhizin	C_22_H_22_O_10_	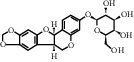
TH11	61358-52-9	Multiflorin A	C_29_H_32_O_16_	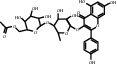
TH12	52657-01-9	Multiflorin B	C_27_H_30_O_15_	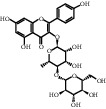
TH13	88515-58-6	Rosamultin	C_36_H_58_O_10_	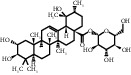
TH14	27215-04-9	Meratin	C_27_H_30_O_17_	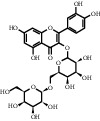
TH15	55804-65-4	Coumarin 343	C_16_H_15_NO_4_	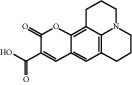
TH16	7786-61-0	2- Methoxy-4-3- vinylphenol	C_9_H_10_O_2_	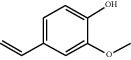
TH17	96-76-4	2,4-Di-tert-butylphenol	C_14_H_22_O	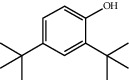
TH18	28564-83-2	2,3-Dihydro-3,5-dihydroxy-6-methyl-4H-Pyran-4-one	C_6_H_8_O_4_	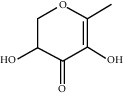
TH19	30692-16-1	5-Tridecanone	C_13_H_26_O	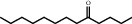
TH20	27147-71-3	2-Methylhexadecane	C_17_H_34_O_2_	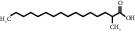
TH21	541-02-6	Decamethylcyclopentasiloxane	C_10_H_30_O_5_Si_5_	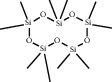
TH22	4410-31-5	Mandelamide	C_8_H_9_NO_2_	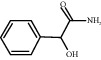
TH23	769-68-6	2-Phenylbutyronitrile	C_10_H_11_ N	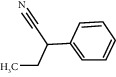
TH24	73-03-0	Cordycepin	C_10_H_13_N_5_O_3_	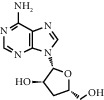
TH25	529-80-6	Multiflorine	C_15_H_22_N_2_O	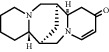
TH26	124-19-6	Nonanal	C_9_H_18_O	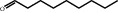
TH27	287100-87-2	n-Hexadecanoic acid	C_16_H_32_O_2_	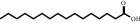
TH28	65-85-0	Benzoic acid	C_7_H_6_O_2_	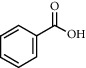
TH29	15356-74-8	Dihydroactinidiolide	C_11_H_16_O_2_	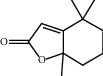
TH30	84-74-2	Dibutyl phthalate	C_16_H_22_O_4_	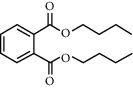
TH31	84-69-5	Diisobutyl phthalate	C_16_H_22_O_4_	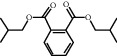
TH32	56221-91-1	13-Tetradecen-1-ol acetate	C_16_H_30_O_2_	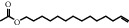
TH33	5129-60-2	Methyl 14-methylpentadecanoate	C_17_H_34_O_2_	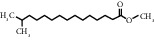

**Table 2:** Molecular docking analysis of active ingredients of peach blossom and core targets.

Targets	PDB ID	Active ingredients	Affinity (kcal/mol)
ESR1	6VIG	Naringenin	−5.63
VEGFA	1MKK	Benzyl beta-D-glucopyranoside	−5.05
PTGS2	5F19	Diisobutyl phthalate	−4.9
PTGS2	5F19	Trifolirhizin	−4.73
ESR1	6VIG	2-Phenylbutyronitrile	−4.57
ESR1	6VIG	Aromadendrin	−4.4
EGFR	5UG9	Cordycepin	−4.2
ESR1	6VIG	(-)-Catechin	−3.99
GSTP1	3GUS	Multiflorin A	−3.12
GSTP1	3GUS	Kaempferol-3-O-rutinoside nicotiflorin	−2.9
GSTP1	3GUS	Multiflorin B	−2.19
GSTP1	3GUS	Meratin	−1.1

## Data Availability

The data used to support the findings of this study are included within the article.
